# Stokes’ Second Problem for a Micropolar Fluid with Slip

**DOI:** 10.1371/journal.pone.0131860

**Published:** 2015-07-10

**Authors:** Olivia Ana Florea, Ileana Constanţa Roşca

**Affiliations:** 1 Faculty of Mathematics and Computer Science, Transilvania University of Braşov, Braşov, Romania; 2 Faculty of Product Design and Environment, Transilvania University of Braşov, Braşov, Romania; University of Zurich, SWITZERLAND

## Abstract

In this paper is presented the model of an incompressible micropolar fluid flow with slip using the initial and boundary conditions when the wall velocity is considered depending on the frequency of the vibration. Regarding the boundary conditions of the velocity at the wall, we remark that there is a discontinuity of the velocity at the fluid-wall interface. The solutions for velocity and microrotation with the given conditions are obtained using the method of numerical inversion of Laplace transform.

## Introduction

The theory of micropolar fluids was introduced for the first time by Eringen, [1]. The polar fluids are those fluids that have a non-symmetric stress tensor. A subclass of polar fluids is represented by the micropolar fluids. The micropolar fluids are fluids with microstructure. Physically, the fluids with rigid particles, randomly oriented that are suspended in a viscous medium represent the micropolar fluids. In this case the deformation of particles is neglected. The theory of micropolar fluids has various applications in the chemical industry (i.e. lubricants, liquid crystals, polymeric fluids), in the biomedical industry (i.e. animal blood) and also in medicine (i.e. the synovial fluid of knee, [2]).

Recently Devakar and Iyengar [3], studied Stokes’ first problem for a micropolar fluid, i.e. the fluid flow through a half-space delimited by a flat plate. Initially both the plate and the fluid are at rest. At time *t* = 0+ the plate suddenly starts to slide slowly in its plane with the constant velocity *u*_0_. Stokes’ second problem refers to the case when the velocity of the wall is time-dependent.

In this paper we will refer to the Stokes’ second problem, [4], when the wall is driven in an oscillatory shearing motion. Ibrahem et al. [5] solved a Stokes’ second problem for a micropolar fluid, embedded into a thermal analysis. With this idea in mind, we take into account a slip between the velocity of the fluid at the wall and the speed of the wall *u*_*w*_. Moreover, we will allow a temporal variation of *u*_*w*_ either in cosine or sine form, inspired by the paper by Khaled and Vafai [6], who were able to obtain exact solutions for clear fluids.

Partial slip at a solid boundary may occur in several situations, that can be divided in two categories:

a) in rarefied gas and/or in micro-nanogeometries, when the Knudsen number is in the range 0.001…0.01, in the so called slip regime, when the Navier-Stokes equations are valid, but slip occurs.
b) In the case of rough surfaces, or some coated surfaces (such as Teflon) which resist adhesion.

Problems of reflection and transmission of plane waves at an imperfect boundary of micropolar elastic bodies have been studied by Sharma, [7]. The temporal behaviour of the solutions of micropolar bodies was studied by Marin, [8].

The study of the behaviour of the components of the velocity profiles for the incompressible steady micropolar fluids flows through a porous medium were studied by Mekheimer, [9]. The study of the effect of the induced magnetic field on peristaltic flow of a micropolar fluid is developed in the papers [10, 11].

Concerning the slip conditions for micropolar fluids we can make a short state of the art for the numerical methods that were used in the literature. Therefore, in the paper [3], Devakar and Iyengar studied the Stokes’ first problem for a micropolar fluid. In this paper the analysis was made for time smaller than 5. In our paper where we study the Stokes’ second problem with slip boundary conditions, the numerical analysis is made for values of time greater than 5. Hence, for the values of time equal with 5, the behaviour of the micropolar fluids is similar with those from [3] and the novelty of this section is given by the analysis of the microrotation and the velocity at values of time equal with 10.

Regarding the study of the Stokes’ second problem, it was analysed by Ibrahem [5], but in his paper was considered only the simplified case without slip boundary conditions. In our study, if we consider the simplified case when the motion is without slip the problem is reduced to the one studied by Ibrahem [5] and if we consider simultaneously that the motion is without slip and that the viscosity ratio is neglected the problem is reduced to a simple Newtonian fluid, case analysed by Puri [12].

The analysis of the micropolar fluids with slip was studied by many researchers from the point of view of the unsteady Couette flow [6] or for an oblique stagnation flow [13], but from the point of view of the Stokes’ second problem, this analysis has not been made before, of author’s best knowledge.

## Analysis

The flow in the directions *y* and *z* does not exist, therefore the flow speed at a given point in the considered half space depends only on *y* coordinate and time, such as the velocity field has the form: $Q=\bar{u}(\bar{y},\bar{t})\vec{i}$, where $\vec{i}$ is the unit vector in the *x* coordinate direction. The microrotation field will be in the form: $v=(0,0,\bar{N}(\bar{y},\bar{t}))$[3].

The continuity equations are [1]:

$$\frac{\partial \rho }{\partial \bar{t}}+\text{div}\left(\rho Q\right)=0$$ (1)

For an incompressible micropolar fluid, the governing equations are, [3]:

$$\rho \frac{\partial \bar{u}}{\partial \bar{t}}=(\mu +\kappa )\;\frac{\;\partial^{2}\bar{u}}{\partial {\bar{y}}^{2}}+\kappa \frac{\partial \bar{N}}{\partial \bar{y}}$$ (2)

$$\rho \bar{j}\frac{\partial \bar{N}}{\partial \bar{t}}=\frac{\;\partial }{\partial \bar{y}}\;(\gamma \frac{\;\partial \bar{N}}{\partial \bar{y}})\;-\kappa \;(2\bar{N}+\frac{\partial \bar{u}}{\partial \bar{y}})$$ (3)

where *μ*, *κ* are viscosity coefficients.

We consider the expression of *γ* inertial viscosity coefficients, given by:

$$\gamma =(\mu +\frac{\kappa }{2})\;\bar{j}=\mu \;(1+\frac{\Delta }{2})\;\bar{j}$$ (4)

where $\Delta =\frac{\kappa }{\mu }$ is the material parameter and the microinertia density $\bar{j}$ is a constant in the present study.

Initial and boundary conditions are

$$\bar{u}\;(\bar{y},\bar{t})=0,\;\bar{N}\;(\bar{y},\bar{t})=0,\;\text{for}\;\bar{t}<0$$ (5a)

$$\bar{u}\;(0,\bar{t})-\beta \frac{\;\partial \bar{u}}{\partial \bar{y}}\;(0,\bar{t})=u_{w},\;\bar{N}\;(0,\bar{t})=-n\frac{\partial \bar{u}}{\partial \bar{y}}\;(0,\bar{t})$$ (5b)

$$\bar{u}\;(\infty ,\bar{t})=0,\;\bar{N}\;(\infty ,\bar{t})=0$$ (5c)

where the wall velocity due to the local peristaltic phenomenon ([10, 11]) is taken as:

$$u_{w}=u_{0}\cos(\bar{\omega }\bar{t}),\text{or}\;u_{w}=u_{0}\sin(\bar{\omega }\bar{t})$$ (6)

where $\bar{\omega }$ is the frequency of the vibration of the blood vessel, [14]. Regarding the boundary condition of the velocity at the wall, we remark that there is a discontinuity of the velocity at the fluid-wall interface, quantified by the parameter *β*.

Further, the microrotation parameter *n* is a constant with values between 0 and 1: *n* = 0 represents the case of concentrated particle flows in which the microelements close to the wall are not able to rotate, see Jena and Matkur [15]; the case *n* = 1/2 indicates the vanishing of antisymmetric part of the stress tensor and denotes weak concentrations, according to Ahmadi [16]; the case *n* = 1 is representative for turbulent boundary layer flows, as suggested by Peddieson [17].

We notice that Devakar and Iyengar [3] considered only the case *n* = 0.

Dimensionless variables are introduced next as [3]

$$y=\frac{\rho u_{0}}{\mu +\kappa }\bar{y},\;t=\frac{\rho u_{0}^{2}}{\mu +\kappa }\bar{t},\;u=\frac{\bar{u}}{u_{0}},\;N=\frac{\mu +\kappa }{\rho u_{0}^{2}}\bar{N},\;j={\left(\frac{\rho u_{0}}{\mu +\kappa }\right)}^{2}\bar{j}$$ (7)

and the problem becomes:

$$\frac{\partial u}{\partial t}=\frac{\;\partial^{2}u}{\partial y^{2}}+m\frac{\partial N}{\partial y}$$ (8)

$$\frac{\partial N}{\partial t}=n_{2}\frac{\;\partial^{2}N}{\partial y^{2}}-n_{1}n_{2}\;(2N+\frac{\partial u}{\partial y})$$ (9)

where

$$m=\frac{\kappa }{\mu +\kappa }=\frac{1}{1+\Delta },n_{1}=\frac{\kappa }{\gamma }{\left(\frac{\mu +\kappa }{\rho u_{0}^{2}}\right)}^{2},n_{2}=\frac{\gamma }{\left(\mu +\kappa \right)j}$$ (10)

The initial and boundary conditions become:

$$u\;(0,t)-\lambda \frac{\;\partial u}{\partial y}\;(0,t)=\{\begin{array}{c} \cos\omega t\\ \sin\omega t \end{array}\},N\;(0,t)=-n\frac{\partial u}{\partial y}\;(0,t)$$ (11a)

u(∞,t)=0,N(∞,t)=0 (11b)

where

$$\omega =\bar{\omega }\frac{\mu +\kappa }{\rho u_{0}^{2}},\lambda =\beta \frac{\rho u_{0}}{\mu +\kappa }$$ (12)

Using the Laplace transform we have the following notations:

$$𝓛\left[u\left(y,t\right)\right]\left(s\right)=\hat{u}\left(y,s\right);𝓛\left[N\left(y,t\right)\right]\left(s\right)=\hat{N}\left(y,s\right);$$

$$𝓛[\frac{\partial u}{\partial y}]\;\left(s\right)=\frac{d\hat{u}}{dy};𝓛[\frac{\partial N}{\partial y}]\;\left(s\right)=\frac{d\hat{N}}{dy};$$

$$𝓛[\frac{\partial u}{\partial t}]\;\left(s\right)=s\hat{u}\left(y,s\right)-u\left(y,0\right)=s\hat{u}\left(y,s\right);$$

$$𝓛[\frac{\partial N}{\partial t}]\;\left(s\right)=s\hat{N}\left(y,s\right)-u\left(y,0\right)=s\hat{N}\left(y,s\right).$$

Taking the Laplace transform with respect to the time, we obtain

$$\frac{\;d^{2}\hat{u}}{dy^{2}}-s\hat{u}+m\frac{d\hat{N}}{dy}=0$$ (13)

$$\frac{\;d^{2}\hat{N}}{dy^{2}}-n_{1}\frac{\;d\hat{u}}{dy}-(2n_{1}+\frac{s}{n_{2}})\;\hat{N}=0$$ (14)

The boundary conditions of the problem Eqs (12 and 13) are

$$\hat{u}\;(0,s)-\lambda \frac{\;d\hat{u}}{dy}\;(0,s)=\frac{1}{s^{2}+\omega^{2}}\{\begin{array}{c} s\\ \omega \end{array}\},\hat{N}\;(0,s)=-n\frac{d\hat{u}}{dy}\;(0,s)$$ (15a)

$$\hat{u}\;(\infty ,s)=0,\hat{N}\;(\infty ,s)=0$$ (15b)

To obtain the general solution of the system of second degree form by the Eqs (13 and 14) it is necessary to reduce it at a system of first degree. For these considerations we will make the following variable change:

$$\frac{d\hat{u}}{dy}={\hat{u}}^{\prime };\;\frac{d\hat{N}}{dy}={\hat{N}}^{\prime }$$ (16)

With the new notations the Eqs (13 and 14) become:

$$\frac{d{\hat{u}}^{\prime }}{dy}=s\hat{u}-m{\hat{N}}^{\prime }$$ (17)

$$\frac{d{\hat{N}}^{\prime }}{dy}=n_{1}{\hat{u}}^{\prime }+(2n_{1}+\frac{s}{n_{2}})\;\hat{N}$$ (18)

The system form by the Eqs (16), (17) and (18) can be written in the matriceal form:

$$\frac{d}{dy}\;(\begin{array}{c} \hat{u}\\ \hat{N}\\ {\hat{u}}^{\prime }\\ {\hat{N}}^{\prime } \end{array})=(\begin{array}{cccc} 0 & 0 & 1 & 0\\ 0 & 0 & 0 & 1\\ s & 0 & 0 & -m\\ 0 & 2n_{1}+\frac{s}{n_{2}} & n_{1} & 0 \end{array})(\begin{array}{c} \hat{u}\\ \hat{N}\\ {\hat{u}}^{\prime }\\ {\hat{N}}^{\prime } \end{array})$$ (19)

Noting with:

$$X\left(y,s\right)=(\begin{array}{c} \hat{u}\\ \hat{N}\\ {\hat{u}}^{\prime }\\ {\hat{N}}^{\prime } \end{array})\;\text{and}\;A\left(s\right)=(\begin{array}{cccc} 0 & 0 & 1 & 0\\ 0 & 0 & 0 & 1\\ s & 0 & 0 & -m\\ 0 & p & n_{1} & 0 \end{array}),$$

where $p=2n_{1}+\frac{s}{n_{2}}$.

The considered system can be written in the reduce form of a homogeneous differential equation:

$$\frac{d}{dy}X\left(y,s\right)=A\left(s\right)\cdot X\left(y,s\right)$$ (20)

The solution of the above equation is:

$$X\left(y,s\right)={\text{e}}^{A(s)y}X\left(0,s\right)$$ (21)

**Proposition 1** *Let be a domain D* ⊂ **C** *an open and connex set, f:D* → **C** *an holomorphic function on D, A = (a_ij_), i, j = 1..4 and I the unit matrix of fourth degree. Taking into account the integral Cauchy formula:*

$$f\left(z\right)=\frac{1}{2\pi i}\int_{\gamma }\frac{f(\zeta )}{\zeta -z}d\zeta =\frac{1}{2\pi i}\int_{\gamma }{\left(\zeta -z\right)}^{-1}f\left(\zeta \right)d\zeta$$

*the Dunfort-Taylor formula for the matrix function f(A) is applied:*

$$f\left(A\right)=\frac{1}{2\pi i}\int_{\gamma }{\left(zI-A\right)}^{-1}f\left(z\right)dz$$

*where (zI − A)^−1^ is the inverse matrix of zI − A, and γ is a closed, simple, smooth curve which contains inside of the delimited domain all eigenvalues of the matrix A.*

**Remark 1** *If the fourth degree matrix A has the distinct eigenvalues z_1_, z_2_, z_3_, z_4_ then the matrix function can be written:*

$$f\left(A\right)=Z_{1}f\left(z_{1}\right)+Z_{2}f\left(z_{2}\right)+Z_{3}f\left(z_{3}\right)+Z_{4}f\left(z_{4}\right).$$ (22)

*The matrix Z_1_, Z_2_, Z_3_, Z_4_ depends on the matrix A only and they will be determined customize the function f hence:*

$$f\left(z\right)=1;f\left(z\right)=z,f\left(z\right)=z^{2},f\left(z\right)=z^{3}$$ (23)

The eigenvalues of matrix *A* are obtained from: *P*(*z*) = det(*A*(*s*) − *zI*) = 0 that is equivalent with the bi-squared equation:

$$z^{4}-\left(p+s-mn_{1}\right)z^{2}+sp=0$$ (24)

with the roots *z*_1_ = −*z*_2_ and *z*_3_ = −*z*_4_. Using the Remark 1 we obtain the following system:

$$\begin{array}{c} I=Z_{1}+Z_{2}+Z_{3}+Z_{4}\\ A=z_{1}Z_{1}-z_{1}Z_{2}+z_{3}Z_{3}-z_{3}Z_{4}\\ A^{2}=z_{1}^{2}Z_{1}+z_{1}^{2}Z_{2}+z_{3}^{2}Z_{3}+z_{3}^{2}Z_{4}\\ A^{3}=z_{1}^{3}Z_{1}-z_{1}^{3}Z_{2}+z_{3}^{3}Z_{3}-z_{3}^{3}Z_{3} \end{array}$$ (25)

with the solutions:

$$\begin{array}{c} Z_{1}=\frac{1}{2}\frac{-z_{1}\cdot z_{3}^{2}\cdot I+z_{1}\cdot A^{2}+A^{3}-A\cdot z_{3}^{2}}{\left(z_{1}^{2}-z_{3}^{2}\right)\cdot z_{1}}\\ Z_{2}=-\frac{1}{2}\frac{z_{1}\cdot z_{3}^{2}\cdot I-z_{1}\cdot A^{2}+A^{3}-A\cdot z_{3}^{2}}{\left(z_{1}+z_{3}\right)\cdot z_{1}\cdot \left(z_{1}-z_{3}\right)}\\ Z_{3}=-\frac{1}{2}\frac{-z_{1}^{2}\cdot A-z_{1}^{2}\cdot z_{3}\cdot I+A^{3}+z_{3}\cdot A^{2}}{z_{3}\cdot \left(z_{1}^{2}-z_{3}^{2}\right)}\\ Z_{4}=\frac{1}{2}\frac{-z_{1}^{2}\cdot A+z_{1}^{2}\cdot z_{3}\cdot I+A^{3}-z_{3}\cdot A^{2}}{z_{3}\cdot \left(z_{1}^{2}-z_{3}^{2}\right)} \end{array}$$ (26)

After some elementary computation we obtain the expression of e^*A*(*s*)*y*^:

$${\text{e}}^{A\left(s\right)y}={\text{e}}^{z_{1}y}Z_{1}+{\text{e}}^{-z_{1}y}Z_{2}+{\text{e}}^{z_{3}y}Z_{3}+{\text{e}}^{-z_{3}y}Z_{4}=E\left(y,s\right)={\left(E_{ij}\right)}_{i,j=\bar{1,4}}$$ (27)

After some algebra, we finally have the solution in the Laplace transformation domain

$$\hat{u}\;(y,s)=E_{11}\hat{u}\;(0,s)+E12\hat{N}\;(0,s)+E_{13}{\hat{u}}^{\prime }\;(0,s)+E_{14}{\hat{N}}^{\prime }\;(0,s)$$ (28a)

$$\hat{N}\;(y,s)=E_{21}\hat{u}\;(0,s)+E_{22}\hat{N}\;(0,s)+E_{23}{\hat{u}}^{\prime }\;(0,s)+E_{24}{\hat{N}}^{\prime }\;(0,s)$$ (28b)

$${\hat{u}}^{\prime }\;(y,s)=E_{31}\hat{u}\;(0,s)+E_{32}\hat{N}\;(0,s)+E_{33}{\hat{u}}^{\prime }\;(0,s)+E_{34}{\hat{N}}^{\prime }\;(0,s)$$ (28c)

$${\hat{N}}^{\prime }\;(y,s)=E_{41}\hat{u}\;(0,s)+E_{42}\hat{N}\;(0,s)+E_{43}{\hat{u}}^{\prime }\;(0,s)+E_{44}{\hat{N}}^{\prime }\;(0,s)$$ (28d)

where

$$\hat{u}\;(0,s)=\lambda {\hat{u}}^{\prime }\;(0,s)+\frac{1}{s^{2}+\omega^{2}}\{\begin{array}{c} s\\ \omega \end{array}\},\hat{N}\;(0,s)=-n{\hat{u}}^{\prime }\;(0,s)$$ (29)

and primes denote differentiation with respect to *y*. We obtain after some algebra the left unknowns ${\hat{u}}^{\prime }\left(0,s\right)$ and ${\hat{N}}^{\prime }\left(0,s\right)$ in the form

$${\hat{u}}^{\prime }\left(0,s\right)=\{\begin{array}{c} s\\ \omega \end{array}\}\frac{1}{s^{2}+\omega^{2}}\cdot \frac{z_{1}z_{3}\left(z_{1}+z_{3}\right)}{\lambda z_{1}z_{3}\left(z_{1}+z_{3}\right)+nmp+z_{1}^{2}+z_{1}z_{3}+z_{3}^{2}}$$ (30)

$${\hat{N}}^{\prime }\left(0,s\right)=\{\begin{array}{c} s\\ \omega \end{array}\}\frac{1}{s^{2}+\omega^{2}}\cdot \frac{nmp\left(z_{1}z_{3}+s\right)-\left(z_{1}^{2}-s\right)\left(z_{3}^{2}-s\right)}{m[\lambda z_{1}z_{3}\left(z_{1}+z_{3}\right)+nmp+z_{1}^{2}+z_{1}z_{3}+z_{3}^{2}]}$$ (31)

where the first choice corresponds to the cosine form, while the second one to the sine form. The quantities *z*_1_ and *z*_3_ are those roots with negative real parts of the equation. With the relations Eqs (29)–(31) we obtain the solutions for the system Eq (28): the image through the Laplace transform of the velocity *u*(*y*, *s*) and the microrotation *N*(*y*, *s*).

Finally, in order to revert to the physical domain, we have to invert the Laplace transform for the results given by Eq (28). A suitable approach to numerically perform this task is the method presented by Honig & Hirdes [18].

The original of the Laplace transformation for the velocity is given by

$$u\;(y,t)=\frac{1}{2\pi i}\int_{c-i\infty }^{c+i\infty }e^{st}\hat{u}\;(y,s)ds$$ (32)

where *c* > 0 is an arbitrary constant greater than the real parts of the singularities of *f*(*y*, *s*). The Eq (32) exists for Re(*s*) ≥ *c* > 0. Taking *s* = *c*+*iθ* we have *ds* = *idθ* and we get:

$$u\;(y,t)=\frac{1}{2\pi }e^{ct}\int_{-\infty }^{\infty }e^{i\theta t}\hat{u}\;(y,c+i\theta )\;d\theta$$ (33)

The function $\hat{u}(y,c+i\theta )$ can be decomposed into real and imaginary part:

$$\hat{u}\left(y,c+i\theta \right)=\text{Re}\;\hat{u}\left(y,c+i\theta \right)+i\cdot \text{Im}\;\hat{u}\left(y,c+i\theta \right).$$

With the above decomposition the the velocity Eq (33) can be written:

$$u\left(y,t\right)=\frac{1}{2\pi }{\text{e}}^{ct}\int_{-\infty }^{\infty }\cos\theta t\cdot \text{Re}\;\hat{u}\left(y,s\right)-\sin\theta t\cdot \text{Im}\;\hat{u}\left(y,s\right)+$$ (34)

$$+i(\cos\theta t\cdot \text{Im}\;\hat{u}\left(y,s\right)+\sin\theta t\cdot \text{Re}\;\hat{u}\left(y,s\right))d\theta$$ (35)

Due to the parity of the real and imaginary part of the function $\hat{u}(y,s)$ it is observed that the imaginary part from Eq (34) is null. Therefore we have:

$$u\left(y,t\right)=\frac{1}{\pi }{\text{e}}^{ct}\int_{0}^{\infty }\cos\theta t\cdot \text{Re}\;\hat{u}\left(y,s\right)-\sin\theta t\cdot \text{Im}\;\hat{u}\left(y,s\right)d\theta$$ (36)

For *t* < 0 we have *u*(*y*, *t*) = 0 that leads us to:

$$\cos\theta t\cdot \text{Re}\;\hat{u}\left(y,s\right)+\sin\theta t\cdot \text{Im}\;\hat{u}\left(y,s\right)=0$$

Hence the function *u*(*y*, *t*) can be written in cosine form:

$$u\left(y,t\right)=\frac{2{\text{e}}^{ct}}{\pi }\int_{0}^{\infty }\text{Re}\;\hat{u}\left(y,s\right)\cdot \cos\theta td\theta .$$ (37)

Using the develop of a 2*T* periodic function in cosine Fourier series, for any 0 ≤ *t* ≤ 2*T* we have the expression for velocity:

$$u\left(y,t\right)=\frac{2{\text{e}}^{ct}}{T}[\frac{1}{2}\text{Re}\;\hat{u}\left(y,c\right)+\sum_{k=1}^{n^{*}}\text{Re}\;\hat{u}(y,c+i\frac{k\pi }{T})\cos\frac{k\pi }{T}t]$$ (38)

and for microrotation

$$N\left(y,t\right)=\frac{2{\text{e}}^{ct}}{T}\;[\frac{1}{2}\text{Re}\;\hat{u}\;\left(y,c\right)\;+\sum_{k=1}^{n^{*}}\text{Re}\;\hat{u}(y,c+i\frac{k\pi }{T})\cos\frac{k\pi }{T}t]$$ (39)

where n* is chosen as

$${\text{e}}^{cT}\cdot \text{Re}\;u\;(y,c+in^{*}\frac{2\pi }{T})\leq \varepsilon T,$$

the good results for *cT* ∈ [5, 10] are obtained for a range for n* ∈ [50, 5000]. with *ɛ* a level of error, *ɛ* ∈ [10^−10^,10^−6^]. Taking in particular *t* = *T*, it follows

$$u\;(y,t)≅u_{n^{*}}\left(y,t\right)=\frac{2e^{ct}}{t}\;[\frac{1}{2}\hat{u}\left(y,c\right)+\text{Re}\;\{\overset{n^{*}}{\underset{k=1}{\sum }}{\;(-1)}^{k}\hat{u}\left(y,c+\frac{ik\pi }{t}\right)\}]$$ (40)

$$N\;(y,t)≅N_{n^{*}}\left(y,t\right)=\frac{2e^{ct}}{t}\;[\frac{1}{2}\hat{N}\left(y,c\right)+\text{Re}\;\{\overset{n^{*}}{\underset{k=1}{\sum }}{\;(-1)}^{k}\hat{N}\left(y,c+\frac{ik\pi }{t}\right)\}]$$ (41)

We shall discuss further the optimallity of parameter *c*, with reference to the paper by Honig and Hirdes [18]. There are two choices for the optimal parameter *c* = *c*_*OPT*_:

$$c_{OPT}=\frac{1}{2t+T}\ln|\frac{R(n^{*})\delta }{t\cdot u_{n^{*}}(y,2t+T)}|\text{(Durbin}\;\text{method)}$$ (42a)

$$c_{OPT}=\frac{1}{4t+T}\ln|\frac{R(n^{*})\delta }{t[u_{n^{*}}(y,4t+T)-u_{n^{*}}(y,8t+T)]}|\text{(Korrektur}\;\text{method)}$$ (42b)

where

$$R\;(n^{*})\;\delta =t\frac{u_{n^{*}}^{1}(y,T)-u_{n^{*}}^{2}(y,T)}{e^{c_{2}T}-e^{c_{1}T}}$$ (43)

Here $u_{n^{*}}^{1}\left(y,T\right)$ and $u_{n^{*}}^{2}\left(y,T\right)$ are calculated either with the method of Durbin, either with the Korrektur method. Honig and Hirdes [18] found that *c*_1_ = 20, *c*_2_ = *c*_1_ − 2 is a good choice.

## Numerical results and discussions

Besides velocity *u* = *u*(*y*) and micro-rotation *N* = *N*(*y*) profiles, we are also interested in computing the quantities of physical interest, which are the wall shear-stress and the local couple stress at the wall

$$\tau_{w}={\left[\left(\mu +\kappa \right)\frac{\partial u}{\partial y}+\kappa N\right]}_{y=0},M_{w}=\gamma {\left(\frac{\partial N}{\partial y}\right)}_{y=0}$$ (44)

They become, in dimensionless form,

$$C_{f}=\frac{\tau_{w}}{\rho u_{0}^{2}}={\left(\frac{\partial u}{\partial y}+mN\right)}_{y=0},m_{w}=\frac{{\left(\mu +\kappa \right)}^{2}}{\gamma \rho^{2}u_{0}^{3}}M_{w}={\left(\frac{\partial N}{\partial y}\right)}_{y=0}$$ (45)

where *C*_*f*_ is the skin friction coefficient and *m*_*w*_ is a dimensionless form of the local couple stress at the wall.

A point we think to deserve attention is about the (dimensionless) times considered by Devakar and Iyengar [3], but also by Helmy [19] and Helmy et al. [20], that are not very large. For instance, Devakar and Iyengar [3] took in their numerical runs dimensionless times less than a value of 5. Certainly, resolution of the problem for larger values of time will require an optimization of the numerical scheme [2].

**Fig 1:**
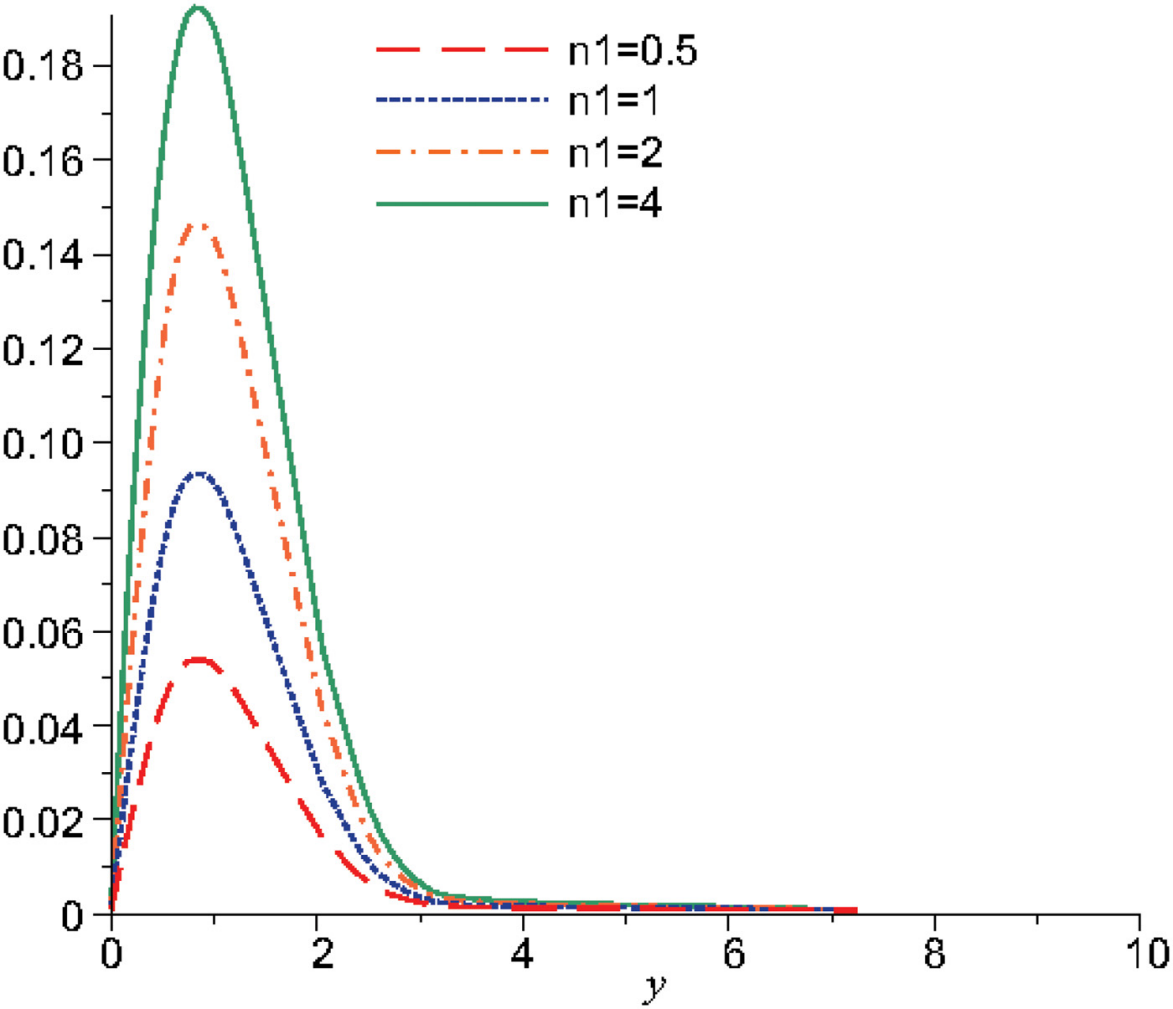
The variation of the microrotation function, *N*, for different values of *n*_1_ when *t* = 10, *m* = 0.5, *n*_2_ = 0.5, *λ* = 0.3.

**Fig 2:**
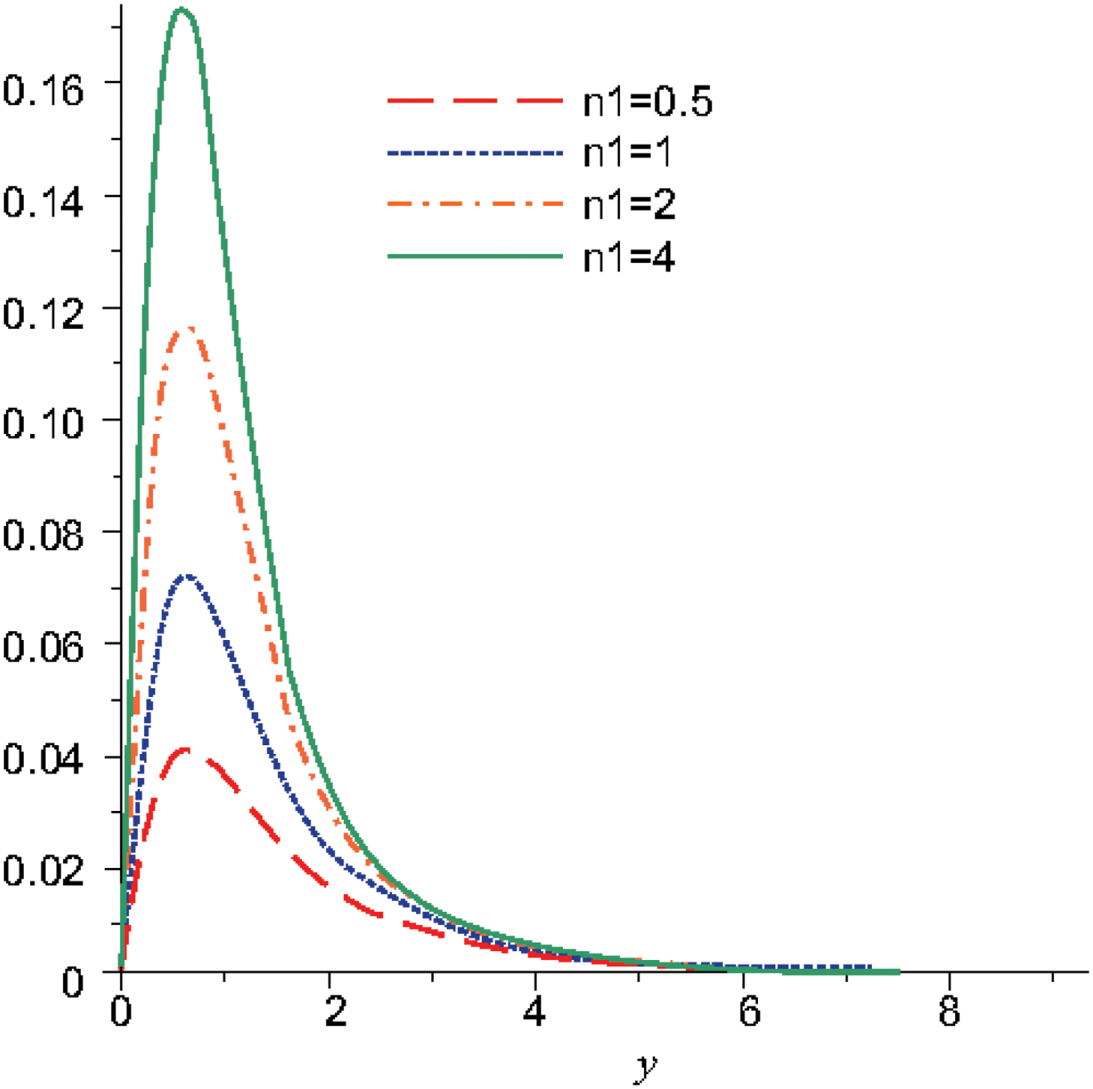
The variation of the microrotation function, *N*, for different values of *n*_1_ when *t* = 5, *m* = 0.5, *n*_2_ = 0.5, *λ* = 0.3.

**Fig 3:**
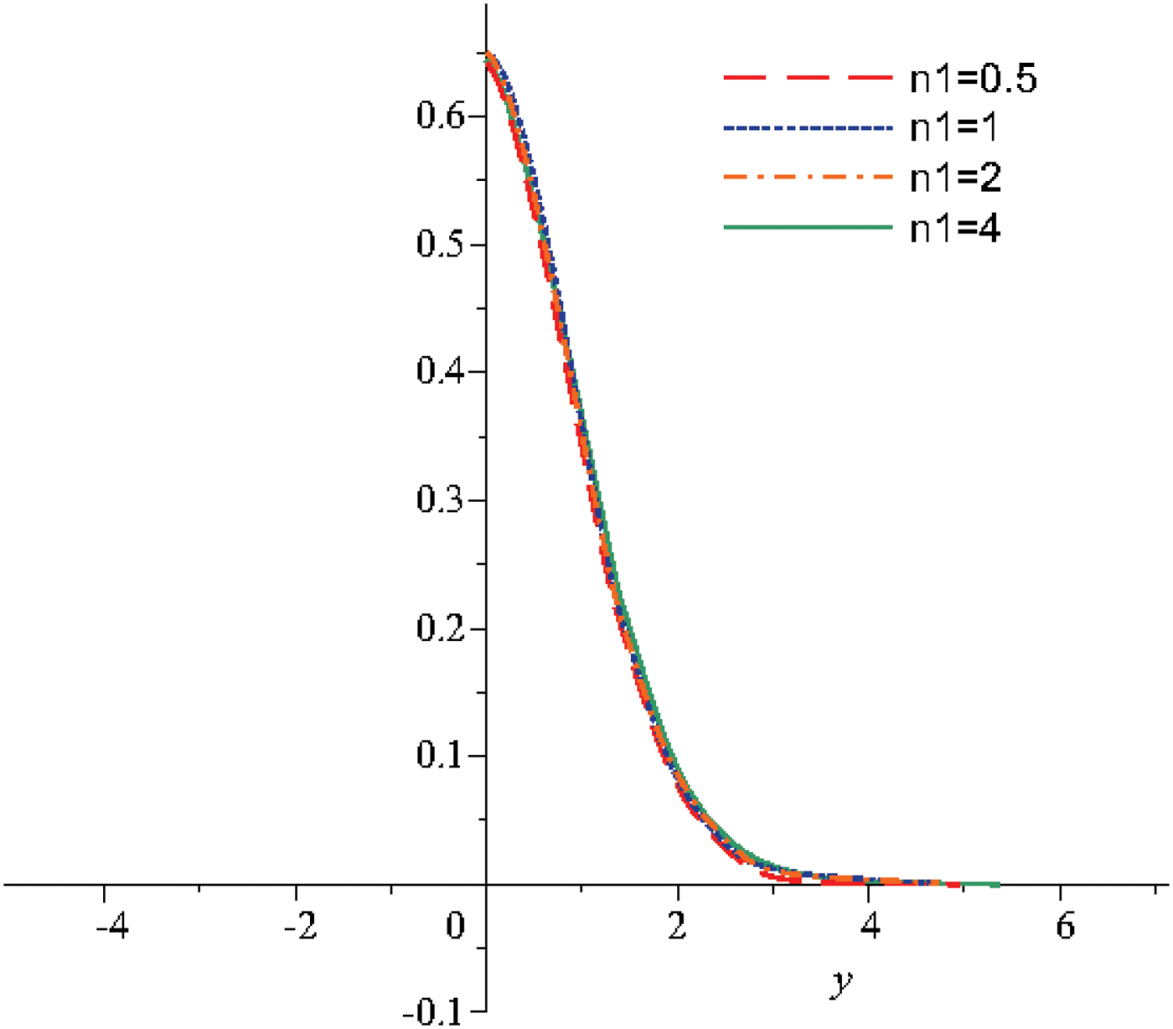
The variation of the velocity function, *u*, for different values of *n*_1_ when *t* = 10, *m* = 0.5, *n*_2_ = 0.5, *λ* = 0.3.

**Fig 4:**
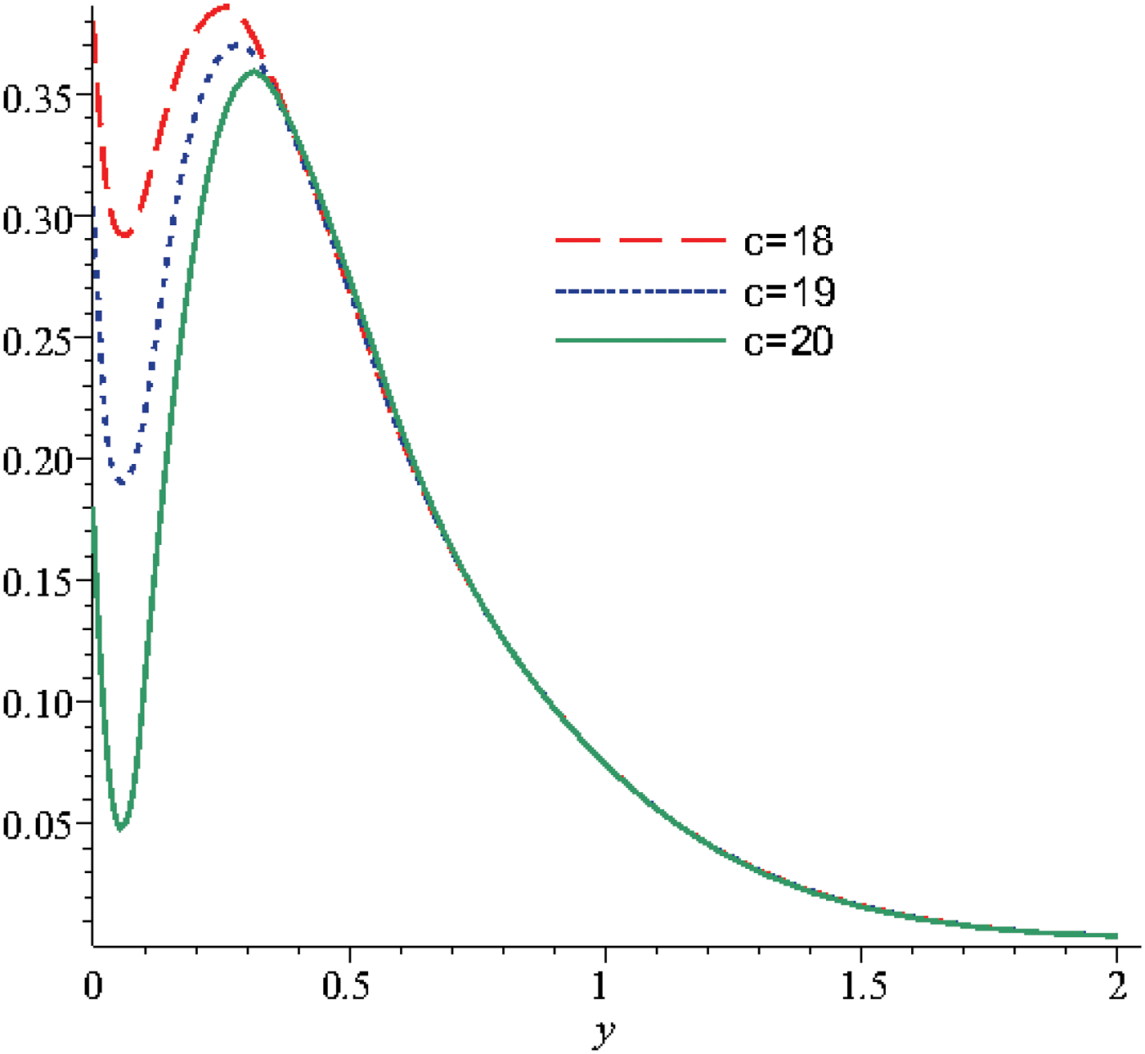
The variation of velocity *u* function of the parameter*c* when *t* = 5.

**Fig 5:**
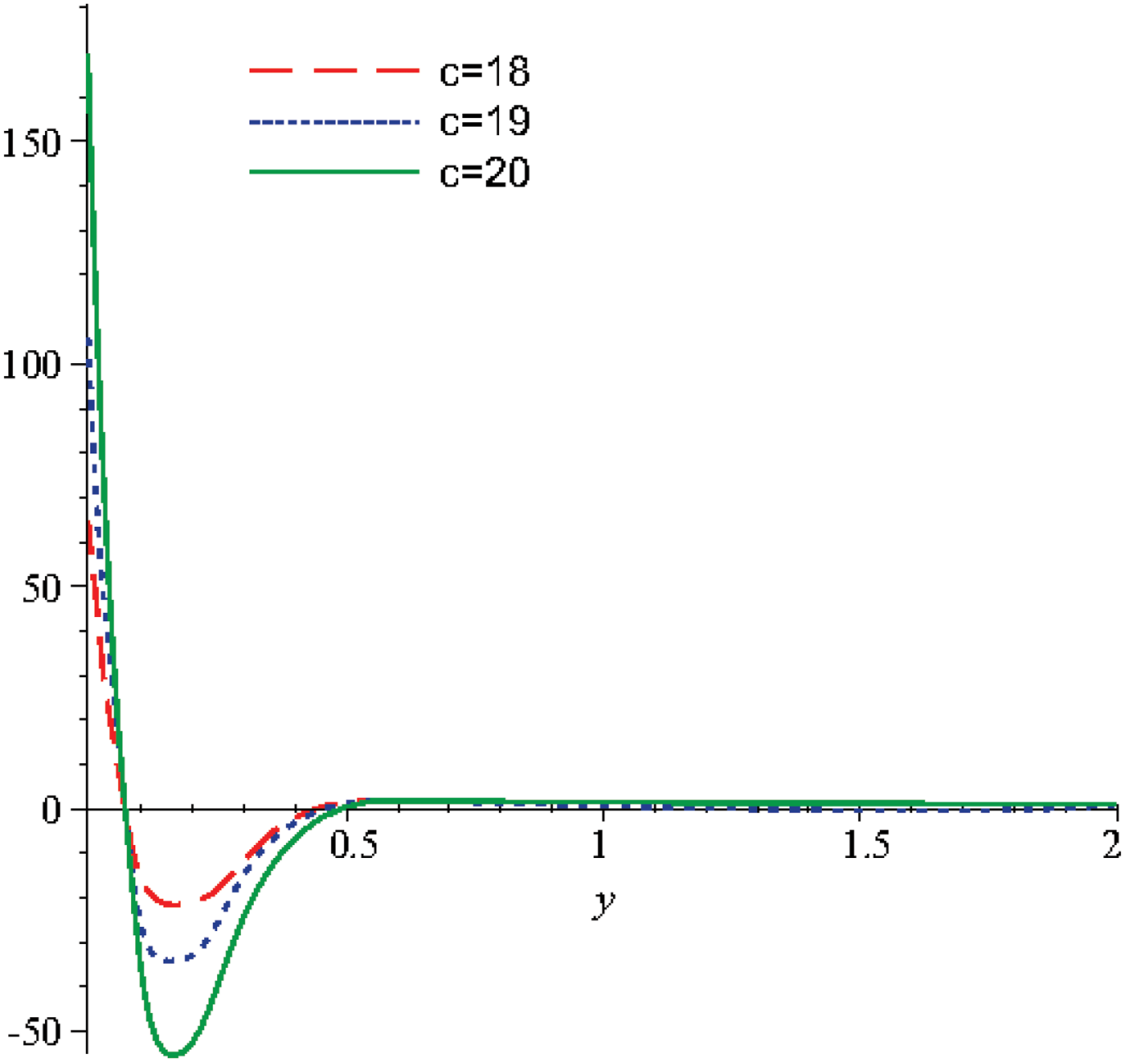
The variation of the microrotation *N* function of the parameter *c* when *t* = 5.

**Fig 6:**
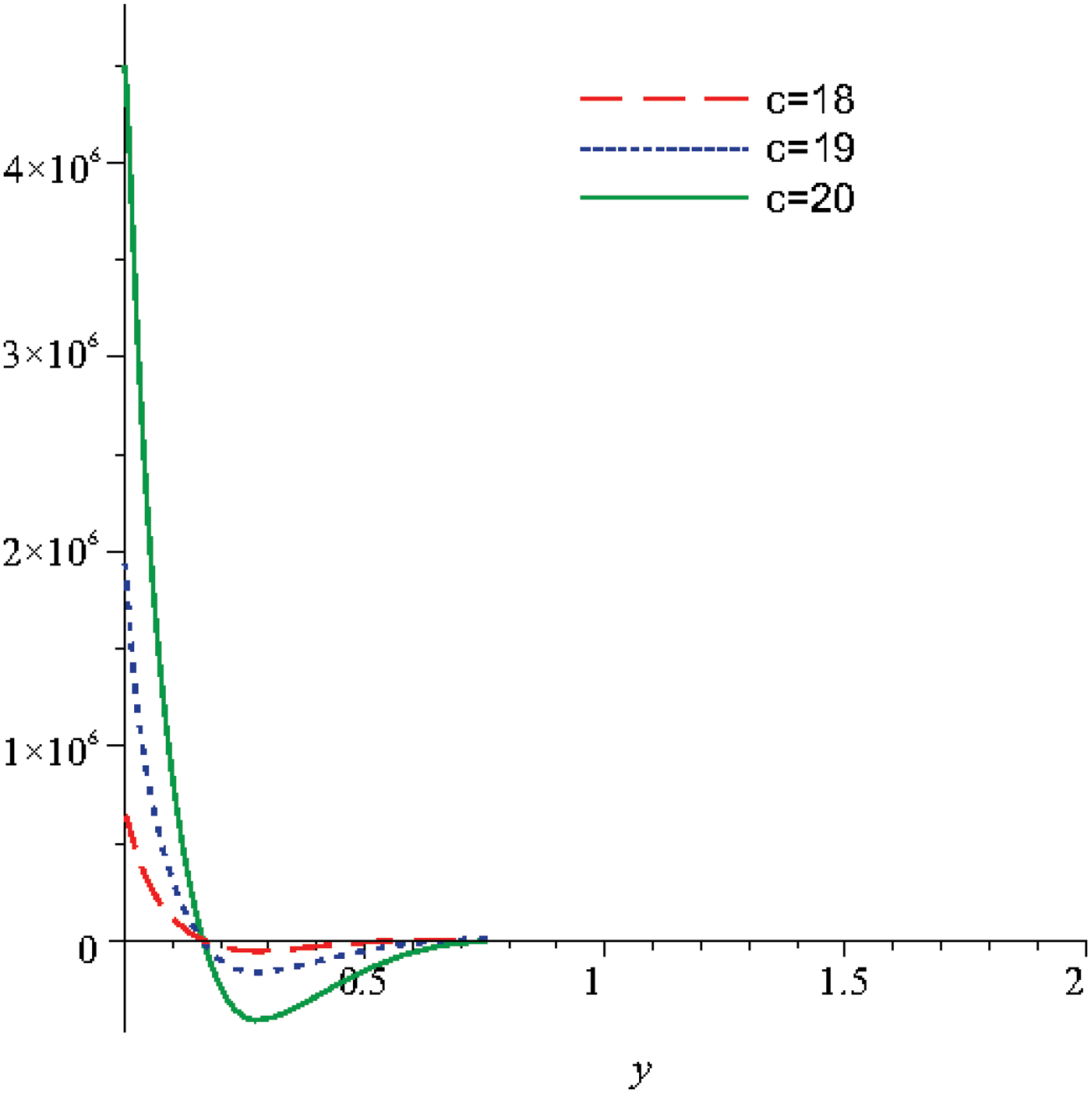
The variation of the microrotation *N* function of the parameter *c* when *t* = 10.

Numerical results are displayed graphically in Figs 1, 2 and 3 for several combinations of the parameters *n*_2_, *m*, *λ* for different moments of time: *t* = 5, *t* = 10, respectively. An overall view of these figures shows that the behaviors of *N* and *u* present a tendency of stabilization. The Figs 4, 5 and 6 show that the choice of *c* becomes stable for dimensionless time:

- greater than 1.7, for *u*
- and even 0.45 for *N*.

The graphical representations of *u* and *N* are in conjunction with the deterioration of *c*_*w*_ and *m*_*w*_ starting with a value of time *t* = 0.5.

**Fig 7:**
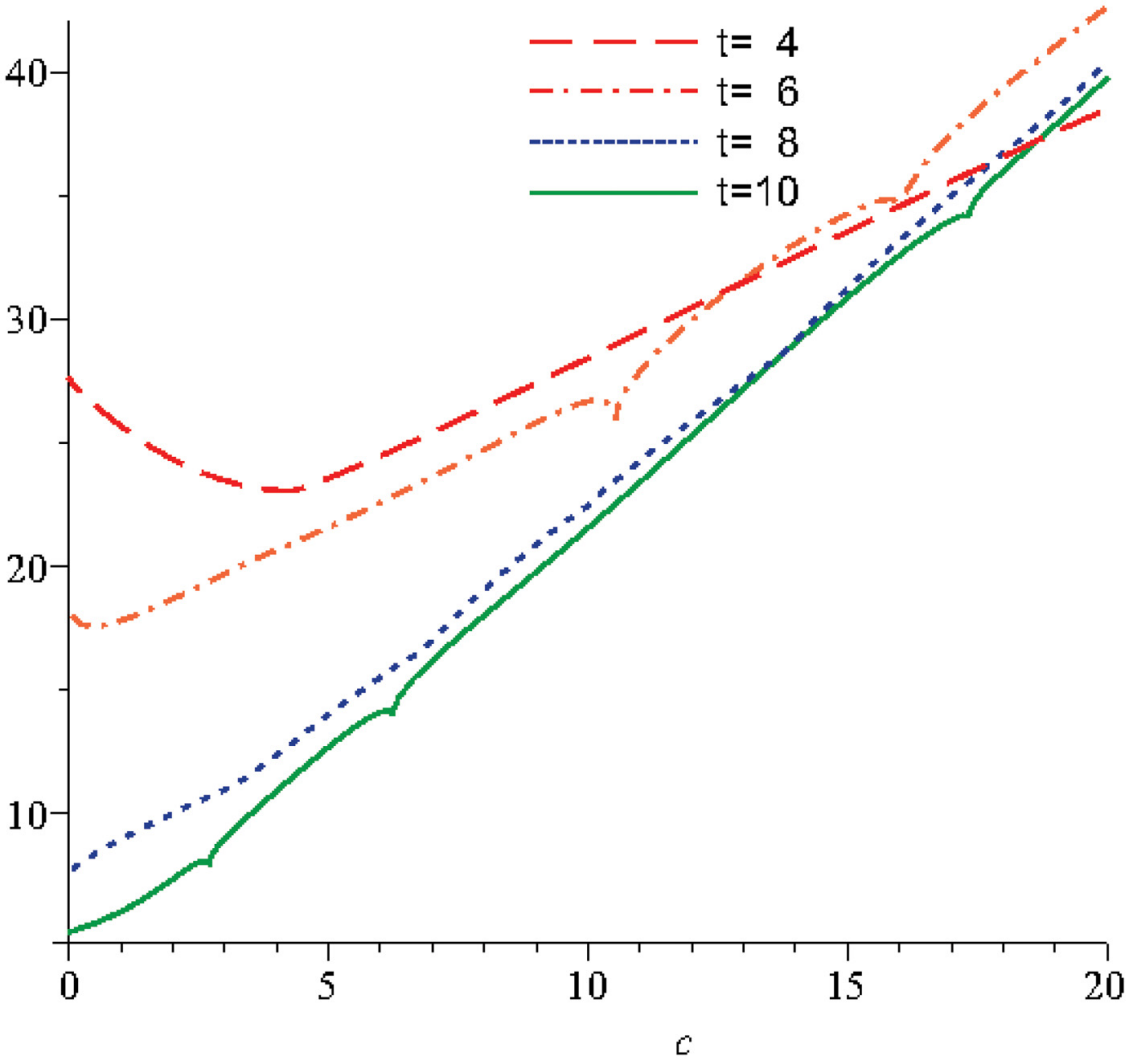
The variation of *c*_*OPT*_ with Durbin method.

**Fig 8:**
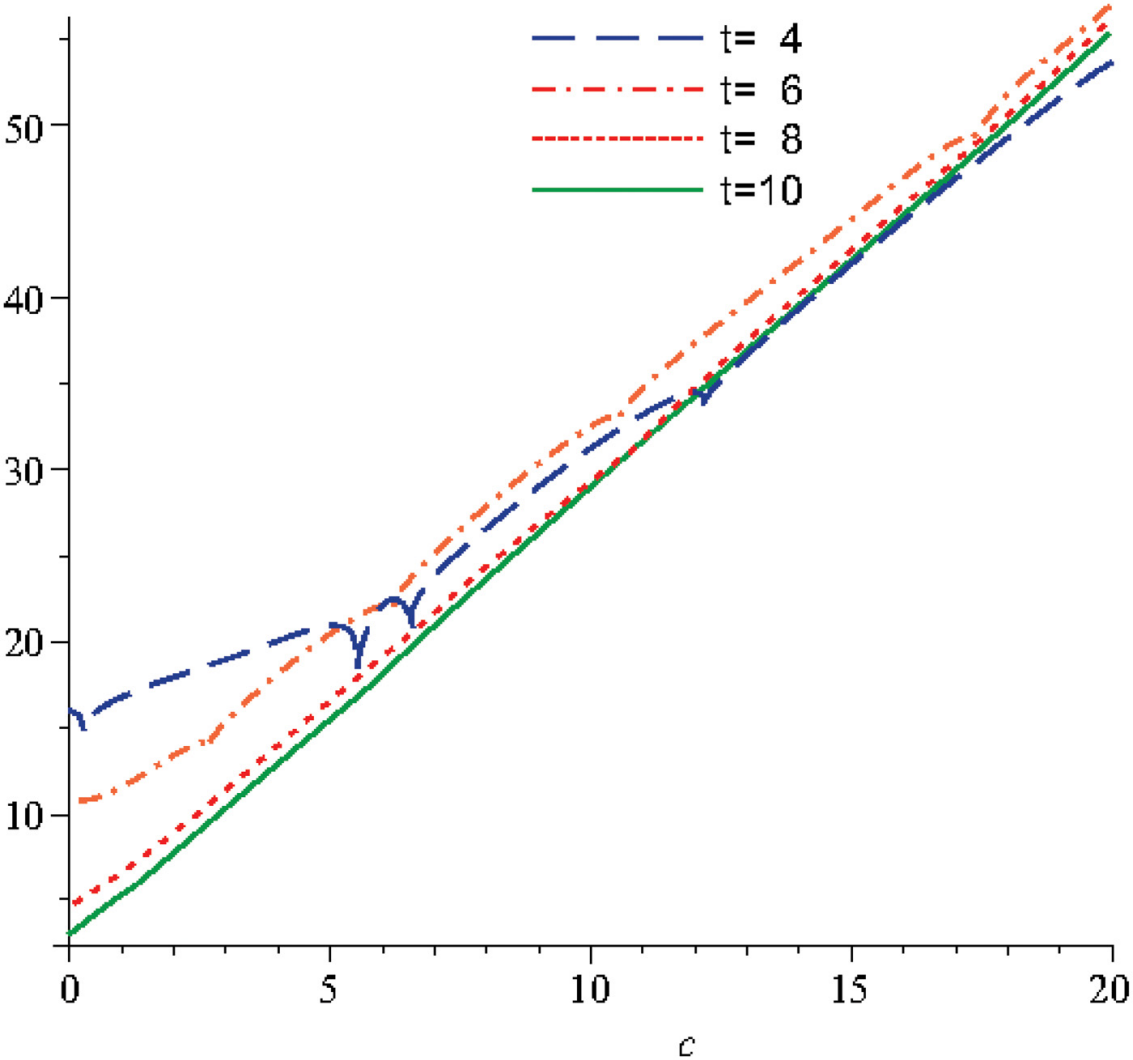
The variation of *c*_*OPT*_ with Korrectur method.

The numerical analysis, with the two mentioned methods: Durbin and Korrectur for different moments in time, highlights that the optimality of parameter *c* is in the range 18..20, see Figs 7 and 8. In this way we observe that the solution of the Stokes’ second problem for a micropolar fluid with slip is stable.

In a further research it will be interesting to approach the same problem through with an adaptation of the existence range of the *c*_*OPT*_ parameter and not of a exact value.

Numerical results are used to simulate the variation of the velocity *u* and of the microrotation *N*, that are graphically represented for different values of the parameters *m*, *n*_1_, *n*_2_ from Eq (10) when the discontinuity parameter *β* varies. The moments of time were chosen at value *t* = 5 for a comparing with Devakar and Iyengar results, [3], and for a value of time greater than 5 that was the last value of time considered by them, [3]. We consider next *t* = 10.

In the Figs 1 and 2 is represented the variation of the microrotation function for different values of the parameter *n*_1_ when the other parameters are fixed. The value of the parameter *m* was considered equal with 0.5 that is equivalent with the particular case when the viscosity coefficients are the same: *μ* = *κ*. The microinertia parameter *j* was considered 3.5, which implies that the inertial viscosity coefficient is $\gamma =\frac{3}{2}$. The amplitude of the velocity initial condition is *u*_0_ = 1, hence the discontinuity parameter *β* becomes function of the microrotation parameter *n*_1_: $\beta =\lambda \sqrt{\gamma \cdot n_{1}}$. In the table 1 are given the values for the parameters *n*_1_ and *β*:

**Table 1:** The values of the microinertia parameter *n*_1_ and the discontinuity parameter *β*.

*n*_1_	*β*
0.5	0.396
1	0.56
2	0.79
4	1.12

The microrotation reaches a maximum for the distance *y* = 0.8, therefore, for *y* ∈ [0,0.8] the function increases and for *y* > 0.8 the function decreases. The microrotation increases as *n*_1_ and, obviously, the discontinuity parameter *β* increase.

The stability of the microrotation occurs for *y* = 3.4 in the case when *t* = 10 (Fig 1) and for *y* = 5.2 in the case when *t* = 5 (Fig 2). Therefore, the installing of the stability state is produced after the initial wave front, that is propagated at distances increasingly smaller, as the time decreases from the motion start.

The Fig 3 shows the variation of the velocity in report with the distance *y* for different values of the parameter *n*_1_ that is chosen as a function of the discontinuity parameter *β*. The simulation is performed in the case when *t* = 10. Hence, it is observed that the velocity decreases as the distance increases. The velocity becomes asymptotically stable for a distance *y* greater than 2.5. The profile curves of the velocity are the same with the studies from Devakar and Iyengar, [3], in case of the Stoke’s first problem of a micropolar fluid flow over a moved plate and also from Mekheimer, [9], in case of the flow of a micropolar fluid through a porous medium.

The numerical study was continued for the variation range of the parameter *c* between the values 18 and 20, in order to obtain the variation of the velocity and of the microrotation, see Figs 4, 5 and 6.

In the Fig 4 the velocity initially decreases and reaches a minimum for *y* ∈ [0.06;0.08], later the velocity reaches a maximum for *y* ∈ [0.25;0.36] and as the distance increases the solution becomes asymptotically stable.

In the Figs 5 and 6 for the considered values of time, we can observe that the microrotation decreases and reaches a negative minimum, later for values of the distance *y* greater than 0.25 the microrotation increases and reaches a positive maximum for *y* ∈ [0.5;0.8] and later as distance increases the solution is stabilizing. To sum up, the stabilization state occurs approximately in the same conditions as in the case when these two functions (velocity and microrotation) are analyzed function of the the optimization coefficient and in report with the discontinuity coefficient, analysis obtained in the above figures.

The analysis of the coefficient *c* according to Durbin and Korrectur leads to the two solutions Eqs (42a) and (42b). For both situations the variation of the optimal values of the coefficients for the approximation solution has the same profile, the differences consist only in the absolute values, see Figs 7 and 8. From here, it results that we can use any method to obtain the optimal coefficient, function of the specific study case. In this situation we can mention that the choice can be imposed by the variation of the other parameters involved in this study.

For the singular case of *λ* = 0 and *n*_1_ = 0 the problem is reduced to the Stokes’ second problem for micropolar fluids with no-slip boundary condition and our results agree with those of Ibrahem [21].

For the singular case of *λ* = 0, *n*_1_ = 0 and *m* = 0 the problem is reduced to a simple Newtonian fluid and our results agree with those of Puri and Kythe [12].

## Conclusions

In this paper we have presented a theoretical approach to study the Stokes’ second problem for micropolar fluids where the novelty are the slip velocity boundary conditions. The proposed model could be a starting point to better understand the mechanism of the fluids’ behavior that belong to the micropolar fluid class, like blood or synovial fluid of knee.

Based on the analysis given above we can draw the following conclusions:

- the velocity in the case of micropolar fluid flow with slip boundary conditions decreases in comparison with the Newtonian fluid flow when the velocity increases;
- the condition that the velocity component at infinity goes to zero gives the stability effect. Due to the fluid’s viscosity, with the increasing of the distance from the wall to the considered point in movement, the half-plane *y* > 0, we assist to a damper of the effect of the wall’s movement, as for *y* → ∞, *u*(*y*, *t*) → 0;
- the microrotation increases as the microrotation parameter and the discontinuity parameter increase;
- the microrotation’s stability occurs behind the initial wave front for distances that tend to infinity;
- the choice of the optimal parameter is independent of the used method, both Durbin as well as Korrectur can be chosen, depending on the parameters involved in the proposed model.
